# On the Application of Pneumatic Chemistry to the Cure of Diseases

**Published:** 1799-04

**Authors:** 


### *On the Application of Pneumatic Chemiſtry to the Cure of Diſeaſes:—By Citizen* FOURCROY.

THIS memoir, which we announced in our laſt, is only an introduction to what the learned author intends to publiſh ſeparately, from time to time, on all parts of the animal economy, on which modern chemiſtry has begun to throw any light; with a deſign to point out what is known of that branch of phyſics, hitherto ſo little advanced, as well as that with which ſome men only pretend to be intimately acquainted; to ſhew what we may hope to learn from experience, and thus, at once, to encourage the labours of other ſcientific men; to cultivate a field, promiſing ſo proſperous a harveſt, and to confound thoſe enemies, who attack the ſyſtem with their diſcordant clamours.

The general reſult which Citizen FOURCROY has always inſiſted upon, is this—that in the new method of operating and reaſoning, adopted by chemiſts, there are contained a very precious and valuable means. Hence it has been already diſcovered, that the blood is heated in reſpiration; that it loſes the carbon and hydrogen; that it abſorbs the oxygen; that it conſequently is renewed, and acquires that quality by which it ſtimulates the heart; that it every where generates heat and life, and changes its nature through the circulation, &c. It has produced a number of other diſcoveries reſpecting reſpiration; the formation of the bile; the nature of albuminous, gelatinous, and fibrous humours. It promiſes greatly to aſſiſt the phyſiologiſt in the ſtudy of the nature and the functions of animals; but, for this purpoſe, an ardent continuance of reſearch is indiſpenſable; as, what has already been diſcovered, is but a ſmall part of what is neceſſary to form a general theory of *animalization*, and the phenomena of the animal economy.

This method is alſo applicable in the knowledge of diſeaſes; but here ſtill leſs has been done than in the former part, and it will be impoſſible to form a doctrine of pathology, until the termination, or at leaſt, a conſiderable progreſs has been made in a work which has yet been ſcarcely begun. One of the objects of theſe applications, which ſeems fartheſt advanced, at leaſt as appears by the noiſe it has lately made among medical men and philoſophers, and the diſcuſſions it has received, in all the ſocieties employed in medical reſearch and ſtudy, is that which regards the *medical properties of* OXYGEN; a word (ſays Citizen Fourcroy), the very name of which will draw upon me numerous groups of men, animated by different opinions and paſſions, but alike enemies to this ſyſtem.

To oppoſe theſe formidable bodies of antagoniſts, Citizen Fourcroy proceeds to ſhew the progreſs of the pneumatic doctrines, from the year

 1779

1779 and 1780, ftating the principles on which it is founded, and the ftrong arguments in its favour. For this detail, we refer the reader to the memoir itfelf, containing 55 pages, 8vo, and fhall content ourfelves with the following extract:—

"A remarkable epoch in the annals of hiftory, that of the war of French liberty, furnifhed me with frequent opportunities of making a ufeful application of my ideas of the healing powers of oxygen. In that war, fo terrible at home, and at the fame time fo glorious for the Republic abroad, a concurrence of circumftances rendered mercury very fcarce. The advice which I then gave to Government, as to the poffibility of fubftituting feveral oxygenated fubftances for mercurial preparations, in the treatment of venereal and pforic patients, who required a great quantity of thefe preparations, in the military hofpitals, not having been followed, becaufe the fuperintending officers of health in the hofpitals doubted the efficacy of the experiments I propofed, while their confidence in mercurial remedies was founded on long experience, I determined to publifh my opinions, and to explain my ideas in my public lectures; confident that they would gradually take root in the minds of the pupils, and would there find that reception and attention, which alone could make them productive of thofe ufeful effects of which I thought them fufceptible. In the fourth year of the Republic, therefore, both in the fchool of medicine, and the mufeum of natural hiftory, I infifted more ftrongly on this doctrine, and the benefits likely to refult from it in the healing art, than I had hitherto done. I particularly adopted, as the fubject of difcuffion, the citric ointment, of which, I knew, there was a great confumption for patients in the itch. I demonftrated, that the oxygenation of fat, by the oxyde of mercury, and the acid of nitre, was to be confidered as the principal caufe of its virtues, and that it might perhaps be poffible, altogether, to difpenfe with mercury in that preparation; that the nitric acid alone appeared to reduce the fat to that ftate of oxydation, where it acquired its well-known medical properties, and there was every reafon to think, that in this ftate, it poffeffed, without mercury, every property of the citric ointment. Citizen ALYON, who was prefent at this lecture, immediately entered into my ideas, and informed me, that he was determined to make the experiment, in order to afcertain the oxygenating effect of the nitric acid upon fat, and to fatisfy himfelf of the properties which it acquired. Thefe firft experiments, made with the good fenfe and prudence of which I knew him poffeffed, were fuccefsful beyond his expectations.—He proved, that the oxygenated fat was antipforic, and antifyphilitic. He added the ufe of the nitric acid, employed with the fame view by fome Englifh phyficians, after their countryman, tryman, SMYTH,* who had firſt made the diſcovery in the Eaſt-Indies. The ſucceſs of this combined method of applying it, externally and internally, was not afterwards diminiſhed, and the report of the commiſſion which had been directed by the ſchool of medicine, to proſecute the new experiments, will beſt evince, how far the firſt ideas which I had given were improved by the attention, the abilities, and the perſeverence of Citizen Alyon, who had a number of obſtacles to encounter.

" In the mean time, while my efforts began to produce ſome fruits in France, philoſophers of other countries, far from being idle contemplators of theſe new ideas, adopted and received them with greater eagerneſs than the French phyſicians. If ſome of theſe carried the pretenſions of the modern chemical doctrines too far; if one, for example, already endeavoured to explain by this all the phenomena of the animal economy; if another ſaw in it the means of prolonging life; the greater number, leaving theſe dangerous paths, followed the more certain road of experience. Three phyſicians have already diſtinguiſhed themſelves in the career, which I congratulate myſelf I have opened, although ſome of them have not, on this head, done me the juſtice which I had a right to expect. M. VON HUMBOLDT, of Berlin, ingeniouſly combined the new facts of Galvaniſm, with the efficacy of chemical agents on the organs of living auimals, and thus clearly explained the phenomena of the functions of vegetables and animals. Dr. BEDDOES, an Engliſh phyſician, examined and carefully determined the action of ſeveral elaſtic fluids in diſeaſes. Dr. ROLLO and Mr. CRUICKSHANK, of the ſame country, in ſtudying the ſymptoms of a diſorder almoſt unknown here, and yet more common than is generally believed, *diabetes mellitus*, collected for the better inveſtigating its nature and cauſes, all that the new chemical diſcoveries offered, and was applicable to that ſubject. They obſerved in that diſeaſe a primary affection of the ſtomach, where vegetable food, by a particular attraction, acquired a ſaccharine quality, which was communicated more or leſs rapidly to the urine; produced a ſur-oxygenated ſtate of the whole ſyſtem; and confirmed that ingenious theory, by the ſucceſs of the remedies they employed. Their work, too little known in France, but with which Citizen Alyon propoſes to enrich the French ſchool, is one of the ſcientific monuments which will beſt prove what advantages medicine may derive from chemiſtry.

" Thus the improvement which I have announced, has made ſuch an impreſſion, that there is no further fear of its being checked in its growth. The only obſtacle which can occur to its progreſs, is, that medical revolutionary ſpirit, which may indiſcreetly accelerate it; a dangerous precipitation of

* We preſume, instead of SMITH, it ought to be SCOTT.

of which there already appear too ſtrong ſymptoms in the learned world. That chemical doctrine, the moderate and prudent application of which may renew our knowledge of the animal economy, ſeems already to miſlead minds, in other reſpects enlightened and ingenious. They wiſh to rear an edifice without having collected the materials. It is undoubtedly known, that the animal ſyſtem, in which oxygen is a great agent, may, either through deficiency or exceſs of that vivifying principle, be impaired; that it is at once the primary ſource of heat, irritability, and the vital motion; that its uſe, either externally or internally, in particular diſeaſes, excites in general the action of life; that we ought to admit two claſſes of remedies in this conſideration, the oxygenating and deſoxygenating; that the former increaſe the activity of the whole ſyſtem, heat, circulation, force, and motion; that the latter, on the contrary, diminiſh all theſe natural effects; that frequently, in the evident empiriciſm of good practitioners, which ſupplies the place of certain principles hitherto wanting in the healing art, the medicines which they preſcribe act by one or other of theſe powers, oxygenating or deſoxygenating. But, if ſuch fundamental aſſertions may be reckoned among the number of thoſe truths, which medicine owes to modern French chemiſtry; if that ſalutary art can already promiſe itſelf important aſſiſtance and more certain lights than we had formerly, how many particulars muſt we yet explain; how many important problems remain yet to be ſolved in chemiſtry; how many ſolutions muſt we attend to, before we can abandon the path hitherto followed, and look upon all the ancient foundations as errors and chimeras? We are yet far from poſſeſſing the requiſite data, and that collection of truths neceſſary to form a complete doctrine, a new medical ſyſtem! Scarcely do we know ſome of the phenomena of certain functions in the animal economy; ſcarcely have we made any fortunate applications of the modern pneumatic diſcoveries; and already we begin to draw from thence general inductions on the nature and cauſes of diſeaſes; ſcarcely have we ſketched the analyſis of ſome of the principal humours in a ſtate of health, and already pretend to claſs diſeaſes after the chemical changes of liquids, and to form a humoral noſology. We ſpeak of claſſing diſeaſes according to the exceſs or deficiency of azote, oxygen, or carbon, before the proportion of the principal conſtituents in any particular animal ſubſtance is aſcertained. What *may be*, is confounded with what *is*; mere appearances, given only as ſuch by authors, are laid down as demonſtrated truths. Perſons of ſuch ſanguine ideas, eager to form theories, as general and fleeting as their fancies, will, it is to be feared, by too precipitate an application, and hypothetical reſults, very eſſentially injure a ſcience, which they have not ſufficiently cultivated to employ it with diſcretion and advantage."

